# A *Sox10* Expression Screen Identifies an Amino Acid Essential for *Erbb3* Function

**DOI:** 10.1371/journal.pgen.1000177

**Published:** 2008-09-05

**Authors:** Kristina Buac, Dawn E. Watkins-Chow, Stacie K. Loftus, Denise M. Larson, Arturo Incao, Gretchen Gibney, William J. Pavan

**Affiliations:** 1 Genetic Disease Research Branch, National Human Genome Research Institute, National Institutes of Health, Bethesda, Maryland, United States of America; 2 George Washington University, Washington, D.C., United States of America; 3 Genome Technology Branch, National Human Genome Research Institute, National Institutes of Health, Bethesda, Maryland, United States of America; Harvard Medical School, United States of America

## Abstract

The neural crest (NC) is a population of embryonic stem cells that gives rise to numerous cell types, including the glia and neurons of the peripheral and enteric nervous systems and the melanocytes of the skin and hair. Mutations in genes and genetic pathways regulating NC development lead to a wide spectrum of human developmental disorders collectively called neurocristopathies. To identify molecular pathways regulating NC development and to understand how alterations in these processes lead to disease, we established an N-ethyl-N-nitrosourea (ENU) mutagenesis screen utilizing a mouse model sensitized for NC defects, *Sox10^LacZ/+^*. Out of 71 pedigrees analyzed, we identified and mapped four heritable loci, called *modifier of Sox10 expression pattern 1–4* (*msp1–4*), which show altered NC patterning. In homozygous *msp1* embryos, *Sox10^LacZ^* expression is absent in cranial ganglia, cranial nerves, and the sympathetic chain; however, the development of other *Sox10*-expressing cells appears unaffected by the mutation. Linkage analysis, sequencing, and complementation testing confirmed that *msp1* is a new allele of the receptor tyrosine kinase *Erbb3*, *Erbb3^msp1^*, that carries a single amino acid substitution in the extracellular region of the protein. The ENU-induced mutation does not alter protein expression, however, it is sufficient to impair ERBB3 signaling such that the embryonic defects observed in *msp1* resemble those of *Erbb3* null alleles. Biochemical analysis of the mutant protein showed that ERBB3 is expressed on the cell surface, but its ligand-induced phosphorylation is dramatically reduced by the *msp1* mutation. These findings highlight the importance of the mutated residue for ERBB3 receptor function and activation. This study underscores the utility of using an ENU mutagenesis to identify genetic pathways regulating NC development and to dissect the roles of discrete protein domains, both of which contribute to a better understanding of gene function in a cellular and developmental setting.

## Author Summary

Genome-wide mutagenesis screens provide a valuable tool to identify genes and genetic pathways regulating complex developmental processes. The neural crest is a population of multipotent cells that gives rise to many different tissue and organ systems. Alterations in the pathways coordinating neural crest formation lead to human developmental disorders. To identify genetic components involved in neural crest development, we combined a whole-genome chemical mutagenesis approach with a mouse strain, *Sox10^LacZ/+^*, that marks neural crest progenitors during early embryogenesis. We identified and determined the chromosomal location of four mutant lines that display impaired neural crest patterning. One of the mutant lines identified carries a single amino acid change that is sufficient to alter neural crest development and cause embryonic lethality without impeding upon protein expression, highlighting the importance of the mutated residue for gene function. This study demonstrates the feasibility of mutagenesis screens to identify the molecular players required for neural crest development as well as to dissect protein domain functions.

## Introduction

The neural crest (NC) is a transient population of multipotent stem cells that arises during vertebrate development. Initially specified at the border between neural and non-neural ectoderm at the time of the neural tube closure (embryonic day (E) 8.5 in mice), NC cells undergo epithelial-mesenchymal transition and migrate along divergent pathways to distinct regions of the embryo [1],[2]. During migration and at the target sites, NC cells respond to various intrinsic and extrinsic factors that specify their differentiation program into specific cell types and tissues, such as neurons and glia of the peripheral and enteric nervous system, smooth muscle cells of the heart outflow tract and cranial blood vessels, chromaffin cells of the adrenal gland, melanocytes of the skin as well as bone and cartilage of the craniofacial structures [3]. Given their broad developmental potential and contribution to multiple tissue and organ systems, it is not surprising that defects in genes and genetic pathways regulating NC development result in a wide spectrum of developmental disorders, collectively called neurocristopathies. These include various human skeletal dysmorphology syndromes (Apert syndrome and Beare-Stevenson cutis gyrata syndrome), nervous system diseases (Hirschsprung disease) and pigment disorders (albinism and Waardenburg syndrome) [4]. Mouse models harboring mutations in orthologous genes often mimic these human disorders, thereby providing a valuable system in which to dissect the pathology of neurocristopathies.

One gene associated with neurocristopathies is *Sox10*, a member of the Sox family of transcription factors, which are characterized by an Sry-type high-mobility group DNA-binding domain [5]. *Sox10* expression is initiated in NC stem cells as they emerge from the dorsal side of the neural tube and is maintained in the melanocyte and glial lineages at later stages [6]–[8]. Work in mouse and zebrafish shows that *Sox10* is involved in maintenance of NC stem cells as well as differentiation into specific lineages [9]–[13]. Mutations in *SOX10* have been associated with Waardenburg syndrome type II (WS2; OMIM 193519), a neurocristopathy defined by abnormal pigmentation and hearing loss; Waardenburg syndrome type IV (WS4; OMIM 277580), an auditory-pigmentary disorder accompanied by loss of enteric ganglia (aganglionosis), a feature of Hirschsprung disease [14], [8], [15]–[17]; and PCWH syndrome (peripheral demyelinating neuropathy, central dysmyelinating leukodystrophy, Waardenbrug syndrome, and Hirschsprung disease), a complex neurological disorder that combines features of four distinct syndromes (OMIM #609136) [18]. The NC defects observed in WS4 are recapitulated in *Sox10* heterozygous mice, which are viable and display hypopigmentation and aganglionic megacolon.

To identify and characterize genetic pathways required for NC development, we utilized a whole-genome N-ethyl-N-nitrosourea (ENU) mutagenesis screen in combination with a mouse model heterozygous for a *Sox10* mutation, *Sox10^tm1Weg^* (herein referred to as *Sox10^LacZ^*) [10]. Homozygous disruption of *Sox10* in *Sox10^LacZ/LacZ^* mice results in embryonic lethality due to impaired NC development while heterozygous *Sox10^LacZ/+^* mice are viable. The presence of a reporter gene replacing one allele of the endogenous *Sox10* in these mice allows for visualization of *Sox10^LacZ^* expression at different stages of embryogenesis. This provides a quick and reliable method to screen for dominant and recessive mutations that alter patterning of the NC lineages marked by *Sox10^LacZ^* expression at midgestation (E11.5), such as melanocytes and glia. We analyzed 71 founders and identified four heritable phenotypes that show an altered embryonic *Sox10^LacZ^* expression pattern. One of the mutant lines identified, *msp1*, was characterized by reduction of *Sox10^LacZ^*-expressing cells in the cranial ganglia, cranial nerves and sympathetic chain. A complementation test confirmed that this mutant is a new allele of the receptor tyrosine kinase, *epidermal growth factor receptor 3* (*Erbb3*), which carries an amino acid substitution in the extracellular region of the protein. Although the ENU-induced mutation does not alter expression of the ERBB3 protein, we show that the subtle amino acid change is sufficient to disrupt ERBB3 signaling, leading to impaired NC development and embryonic lethality.

## Results

### ENU Mutagenesis to Identify Mutations Altering *Sox10* Expression Pattern

**Figure 1:**
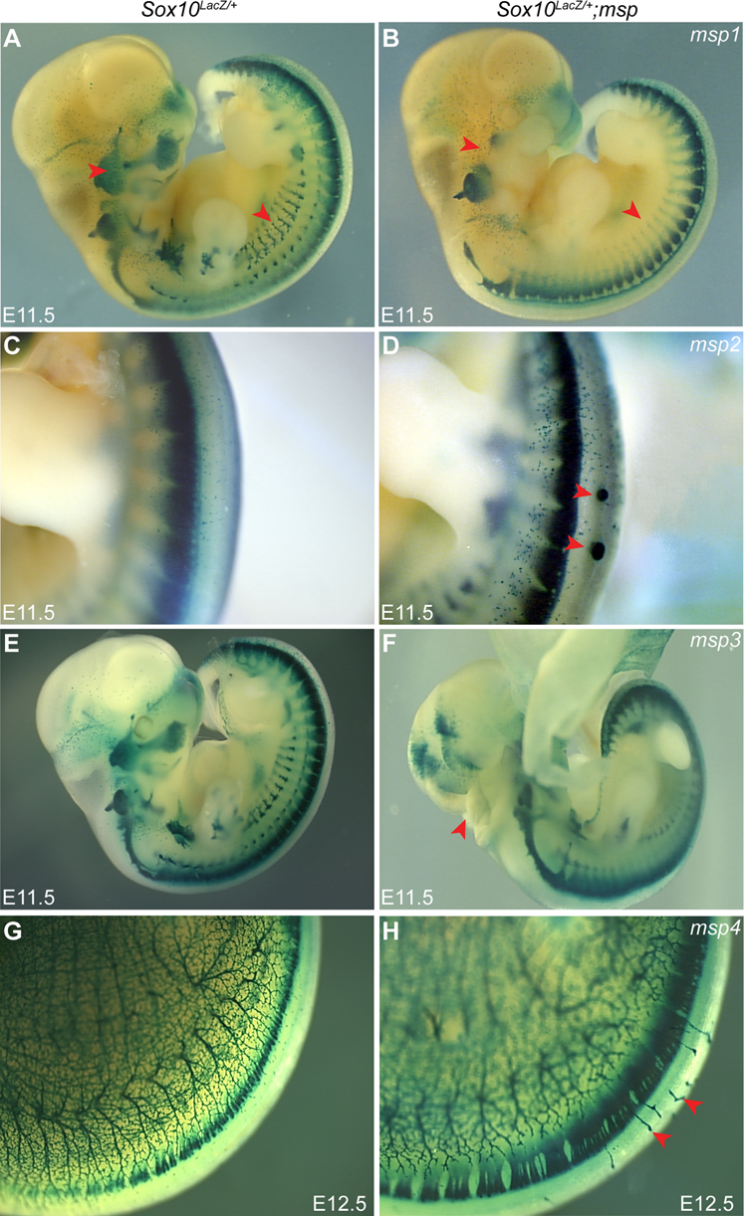
ENU Mutagenesis Screen Identifies Four Embryonic Phenotypes Affecting *Sox10^LacZ^*-Expression Pattern. An age-matched wild-type control embryo (A, C, E, G) is shown next to a representative homozygote embryo for *msp1* (B), *msp2* (D), *msp3* (F), and *msp4* (H). The *Sox10^LacZ/+^*; *msp1^−/−^* embryos show reduction in *Sox10^LacZ^*- expressing cells in the cranial ganglia, cranial nerves and the sympathetic ganglia (B) (red arrowheads). Note the ectopic *Sox10^LacZ^*-expressing masses along the dorsal surface of the neural tube in *Sox10^LacZ/+^*; *msp2^−/−^* embryos (D) (red arrowheads). Neural tube fails to close in *Sox10^LacZ/+^*; *msp3^−/−^* embryos (F) (red arrowheads), thus disrupting normal *Sox10^LacZ^* expression in hindbrain. The *Sox10^LacZ/+^*; *msp4^−/−^* embryos show aberrant *Sox10^LacZ^*-expressing projections (H) (red arrowheads).

To identify genes involved in NC development, we utilized an ENU-mutagenesis screen for mutations that alter the pattern of *Sox10*-expressing NC cells during mouse embryogenesis. In this screen, ENU-treated BALB/cJ males were mated with C57BL/6J females to generate first generation (G_1_) offspring (Figure S1). G_1_ mice were subsequently crossed with *Sox10^LacZ^* ^/+^ heterozygous females, which contain a β-galactosidase reporter gene knocked into the endogenous *Sox10* locus, providing a tool to visualize expression of NC derivatives. Second generation (G_2_) *Sox10^LacZ/+^* female offspring, which show minimal variation in hypopigmentation [19], were then backcrossed to their G_1_ fathers, and E11.5 G_3_ embryos were screened for altered *Sox10^LacZ^* expression. In total, 71 G_1_ males were bred and approximately six litters of G_3_ offspring from each pedigree were analyzed at E11.5 when *Sox10^LacZ^*-expression marks the melanocyte and glial lineages of the NC. Five of the 71 G_1_ pedigrees exhibited an embryonic phenotype with an altered pattern of *Sox10^LacZ^*-expression that was striking in comparison to the normal variation we observe on this mixed genetic background. Five of the 71 G_1_ pedigrees exhibited an embryonic phenotype with altered patterning of *Sox10^LacZ^*-expressing cells. Four of these five phenotypes were reproducible in subsequent generations indicating heritable, Mendelian phenotypes that either directly or indirectly alter the normal pattern of *Sox10^LacZ^*-expression. The four loci were named modifier of *Sox10* expression pattern 1–4 (*msp1*, *msp2*, *msp3*, and *msp4*; Figure 1).

### Chromosomal Localization of *msp* Loci

**Figure 2:**
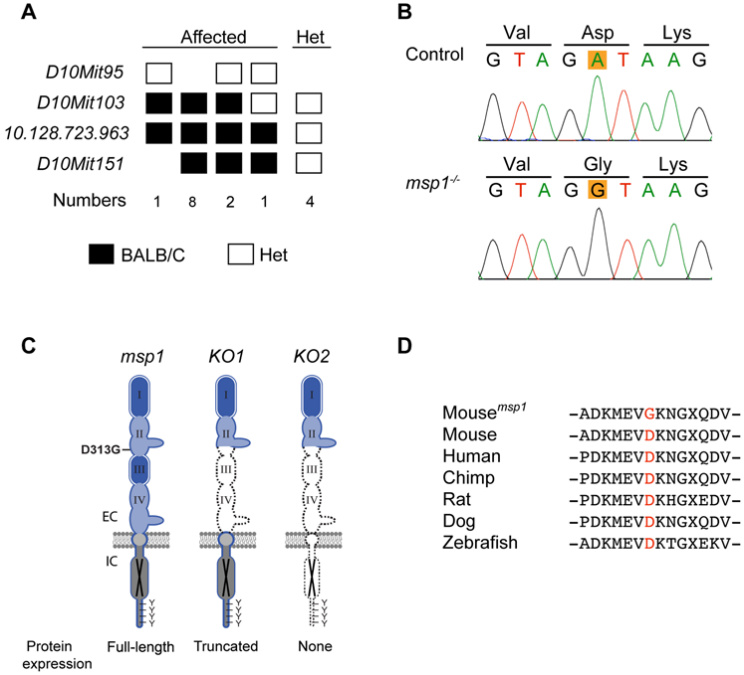
Linkage Analysis and Molecular Characterization of the *msp1* Mutation. (A) Fine mapping of the *msp1* locus on mouse Chr 10. The mutation is confined within a region distal to *D10Mit103*. The number of affected mice analyzed is shown below genotyping data (homozygote black square; heterozygote white square). (B) Sequencing results of *Erbb3* from control and *msp1* embryos. The *msp1* embryos carry an A-to-G transition in exon 8 of the *Erbb3* gene that results in change of the aspartic acid (D) to glycine (G) at position 313. (C) Position of the *msp1* mutation (D313G) in the ERBB3 protein. The ERBB3 protein consists of an extracellular region (EC), a single transmembrane-spanning region, and an intracellular (IC) portion that contains a conserved tyrosine-kinase domain flanked by a carboxy-terminal tail with tyrosine phosphorylation sites. The extracellular region is further subdivided into four domains: domain I, II, III, and IV. Domains I and III are homologous; domains II and IV are homologous. ERBB3 is a kinase-deficient receptor (black X). The D313G is located in the domain II (also known as cysteine-rich region I (CR1)) of ERBB3. In *Erbb3^KO1^* allele a large portion of the extracellular domain is deleted, thus forming a truncated ERBB3 protein. In *Erbb3^KO2^* allele no protein is made due to the insertion of premature termination codons in all three reading frames. (D) Multiple sequence alignment of *Erbb3* from various species. The substituted aspartic acid (D313) is highly conserved among different species.

The four *msp* pedigrees displayed a variety of phenotypes including loss of *Sox10^LacZ^* cells in a subset of NC lineages (*msp1*), ectopic *Sox10^LacZ^*-expressing cell masses disrupting neural tube closure (*msp2*), open neural tube (*msp3*), and aberrant *Sox10^LacZ^*-expressing cells projecting from the dorsal root ganglia (DRG) over the neural tube (*msp4*). A brief overview of each of these phenotypes is presented (Figure 1, Table 1). To characterize these *msp* loci, we first determined their genomic location. For *msp1*, DNA from four affected G_3_ embryos along with three parental G_2_ samples was used for interval haplotype analysis [20]. Genotyping 73 simple sequence-length polymorphic (SSLP) markers spaced throughout the genome, we observed that the *msp1* phenotype consistently segregated with the BALB/cJ allele in a 3 Mb region distal to marker *D10MIT103* on Chr 10 (Figure 2A). To confirm linkage to this region, an additional 38 *Sox10^LacZ/+^* G_3_ embryos were genotyped. All 12 embryos that genotyped as homozygous BALB/cJ for this region were phenotypically affected, indicating that the *msp1* phenotype was fully penetrant. A similar mapping strategy was used to localize *msp2*, *msp3*, and *msp4* to chromosomes 11, 9, and 6, respectively (Table 1). For all four *msp* loci, the region of linkage continued to segregate with the phenotype during six or more generations of outcrossing to C57BL/6J.

**Table 1:** Four embryonic phenotypes identified in ENU screen.

	*msp1*	*msp2*	*msp3*	*msp4*
**Embryonic Phenotype**	partial loss of *Sox10* expression	ectopic *Sox10* expressing cells	open neural tube	disorganized *Sox10* cell migration
**Inheritance**	recessive	recessive	recessive	recessive
**Viability at Weaning** *	Lethal 0/78	Reduced Viability 6/64 (9%)	Lethal 0/54	Reduced Viability 10/166 (6%)
**Markers in Genome Scan**	73 SSLPs	884 SNPs	77 SSLPs	58 SSLPs
**# Samples in Genome Scan**	7	14	12	15
**Chromosome NCBI Bld. 36**	**Chr10** 125.14–129.96 Mb	**Chr11** 58.38–92.88 Mb	**Chr9** 106.27–114.85 Mb	**Chr6** 88.34–92.57 Mb
**Proximal & Distal Marker**	D10MIT103 ter	rs3697686 rs3714299	D9Mit51 D9Mit16	**rs4135401 D6Mit284

*: Number of homozygote mutants observed at weaning from a heterozygote intercross established after outcrossing the G_1_ founder to C57BL/6J for several generations. Expected 25% for viable phenotype.

**: Sequencing revealed a nonsense mutation at nucleotide 2997G>T (NM_008881) in the *Plxna1*gene. Recent data suggest that this mutation is likely to cause the *msp4* phenotype [44], however additional complementation testing is required.

### Embryonic *msp* Phenotypes Are Recessive and Lethal

In each of the four pedigrees, the frequency of affected *msp* embryos in the initial screen was consistent with a recessive mode of inheritance. To confirm this, we analyzed embryos from a cross using a single obligate heterozygote carrier (G_1_ *msp*/+×*Sox10^LacZ/+^*). In all four pedigrees, we failed to observe any affected embryos among the embryos collected (N≥20; P≤0.01). Additionally, as the pedigrees were outcrossed to C57BL/6J, phenotypically affected embryos consistently segregated with homozygosity for BALB/cJ alleles at the linkage regions, thus confirming that the four *msp* phenotypes identified in our ENU screen were recessive. To determine if homozygous embryos were viable, intercrosses were established between heterozygote carriers for each of the four phenotypes. Genotyping of viable mice at weaning age revealed no homozygous viable mice for *msp1* or *msp3* indicating that these phenotypes are fully penetrant with respect to lethality (Table 1). A small number of viable *msp2/msp2* and *msp4/msp4* homozygous mice were observed at weaning, however the frequency was significantly reduced, thus confirming that these two phenotypes are lethal with incomplete penetrance (Table 1).

### *msp1* Carries a Molecular Defect in *Erbb3*

The region of Chr 10 in which linkage was detected for *msp1* contained more than 100 annotated genes. However, we identified the *epidermal growth factor receptor 3* (*Erbb3*) gene as a good candidate because the *Erbb3* null phenotype, which has been previously described, exhibits NC defects similar to those observed in the *msp1* mutants [21],[22]. *Erbb3*, a member of the EGFR receptor tyrosine kinase family, binds to neuregulins (NRGs) and plays an important role in cellular processes during development and cancer progression [23]. To determine whether an ENU mutation in *Erbb3* was responsible for the *msp1* phenotype, we sequenced the coding exons of *Erbb3* from genomic DNA of an affected G_3_ embryo and compared it to the sequence of *Erbb3* in the parental C57BL/6J and BALB/cJ strains. Sequencing of *Erbb3* in the *msp1*embryos revealed only one difference, a point mutation (an A-to-G transversion) at nucleotide 938 (GenBank NM_010153) (Figure 2B), predicting an amino acid change from an aspartic acid to glycine at position 313 (D313G) (NP_034283) (D313 corresponds to D294 in the mature, processed protein). A multiple sequence alignment indicated that D313 is located in domain II (also known as cysteine-rich region 1 (CR1)) of the extracellular portion of the protein (Figure 2C). Comparative sequence analysis revealed that the mutated aspartic acid residue is conserved from humans to zebrafish (Figure 2D), suggesting that this residue might be important for the receptor function.

### *msp1* Is Allelic to *Erbb3*

**Figure 3:**
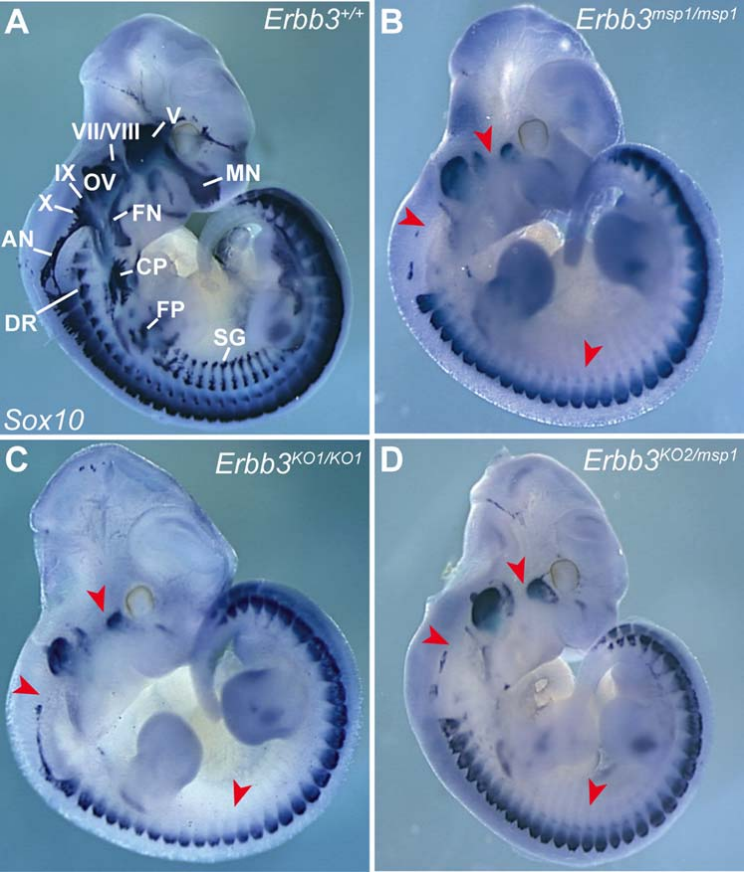
Complementation Test between *msp1* and *Erbb3* Null Allele in Mice. Heterozygous *msp1/+* mice were mated with *Erbb3^KO1^/+* and *Erbb3^K02^/+* mice to generate compound heterozygote embryos. *Sox10* expression was analyzed in control (A), *msp1* (B), null (C), and compound heterozygous embryos (D) at E11.5 by *in situ* hybridization. The mutant genotypes tested showed reduced expression of the *Sox10* transcript in the cranial ganglia, cranial nerves and sympathetic ganglia (red arrowheads). Lack of complementation of *Sox10* expression pattern in the compound heterozygous embryos confirmed that *msp1* is allelic to *Erbb3*. The *Sox10*-expressing structures in the *Erbb3^+/+^* embryo are: maxillary nerve (MN), facial nerve (FN), cervical plexus (CP), forelimb plexus (FP), dorsal ramus (DR), sympathetic ganglia (SG), trigeminal ganglia (V), geniculate ganglia (VII), acoustic ganglia (VIII), superior ganglia (IX), jugular ganglia (X), accessory nerve (AN) and otic vesicle (OV).

To assess if the *msp1* phenotype is due to the point mutation in *Erbb3*, we carried out *in vivo* complementation tests between *msp1* and two previously described *Erbb3* null alleles, *Erbb3^tm1Cbm^* (referred to here as *Erbb3^KO1^*) and *Erbb3^tm2Cbm^* (referred to here as *Erbb3^KO2^*) [22]. In *Erbb3^KO1^* mice a large portion of the extracellular region is replaced with neomycin cassette, leading to production of a truncated ERBB3 protein (∼120 kDa), while no protein is made from the *Erbb3^KO2^* allele due to introduction of the termination codon in the extracellular portion of the gene (Figure 2C). To determine whether *msp1* complements *Erbb3* null alleles, we assayed *Sox10* expression by *in situ* hybridization in compound heterozygous embryos (Figure 3). At E11.5 in *Erbb3^+/+^* embryos, expression of *Sox10* transcript was detected in the cranial ganglia, cranial nerves, sympathetic chain and DRG (Figure 3A). Homozygote *Erbb3^KO2^* (data not shown) and *Erbb3^KO1^* (Figure 3C) showed reduced *Sox10* mRNA-expressing cells in the cranial ganglia, cranial nerves and sympathetic ganglia while exhibiting wild-type *Sox10* mRNA expression in other NC-derived tissues, similar to *msp1* embryos (Figure 3B). *Erbb3^KO1/msp1^* (data not shown) and *Erbb3^KO2/msp1^* compound heterozygous embryos (Figure 3D) also showed reduction in *Sox10*-expressing cells identical to that of *Erbb3^msp1^* homozygotes. These results show that *Erbb3^msp1^* does not complement *Erbb3^KO1^* or *Erbb3^KO2^* phenotypes, demonstrating that the NC defects observed in *msp1* embryos are due to the mutation in *Erbb3*.

**Figure 4:**
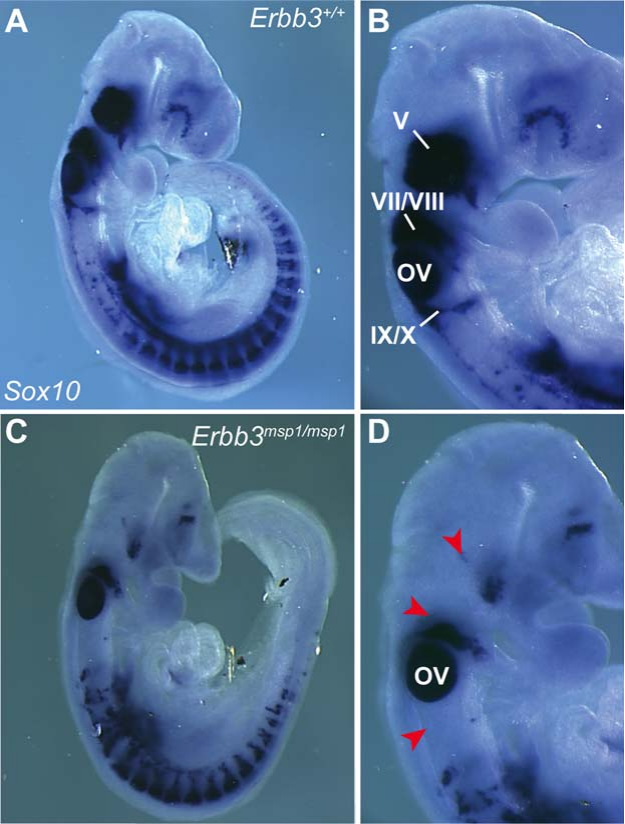
Expression Analysis of Endogenous *Sox10* Transcript at E9.0. In *Erbb3^+/+^* (A, B), *Sox10*-expressing cells are found in the cranial ganglia and DRGs. In *Erbb3^msp1/msp1^* (C, D), *Sox10*-expressing cells are detected in DRG but reduced in the cranial ganglia (red arrowheads). Labeled are trigeminal ganglia (V), geniculate and acoustic ganglia (VII/VIII), superior and jugular ganglia (IX/X) and the otic vesicle (OV).

We further investigated the timing of the NC defects in *Erbb3^msp1/msp1^* embryos by analyzing *Sox10* expression earlier in development. At E9.0 (18 somite stage) *Sox10* expression was already reduced in trigeminal (V), geniculate (VII), and acoustic (VIII) ganglia of *Erbb3^msp1/msp1^* embryos (Figure 4), indicating that the NC defect in the *Erbb3^msp1/msp1^* embryos is evident as early as E9.0. This data are consistent with the timing of NC defects reported for *Erbb3* null alleles [24],[25],[22].

To further compare the phenotypes of *msp1* to null alleles of *Erbb3*, embryonic lethality was assessed in *msp1* embryos. Dissection of embryos generated from several independent *Erbb3^msp1/+^* intercrosses indicated that *Erbb3^msp1/msp1^* embryos were present at expected numbers at E11.5, in accordance with the Mendelian ratio of 25% (Table S1). However, the number of *Erbb3^msp1/msp1^* embryos was dramatically reduced between E12.5 (N = 15, 17%) and E13.5 (N = 6, 8%). No live embryos were observed at weaning stage. These results demonstrate that homozygosity for *Erbb3^msp1^* causes embryonic lethality, with the majority of mutant embryos dying by E13.5. The frequency of weaned heterozygous animals was 65%, indicating there was no lethality associated with heterozygosity. The viability data observed for *Erbb3^msp1^* are consistent with viability data previously shown for *Erbb3^KO1/KO1^* and *Erbb3^KO2/KO2^* animals [22]. Taken together, our data support the hypothesis that even though the *Erbb3^msp1^* allele carries a single amino acid alteration, this point mutation significantly impairs *Erbb3* such that this allele phenotypically resembles *Erbb3* null alleles.

### *Erbb3* mRNA and Protein Are Expressed in the *msp1* Embryos

**Figure 5:**
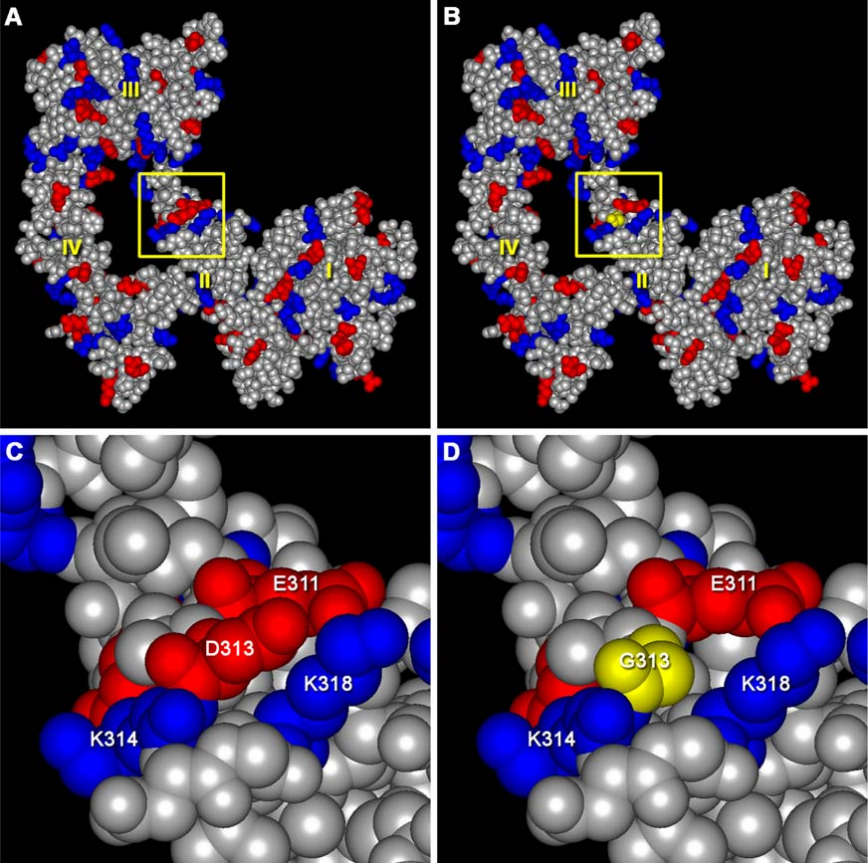
Modeling the *msp1* Mutation. Structural models of mouse wild type (A) and *msp1*-mutant (B) ERBB3 extracellular domains in the “closed” conformation. Negatively charged residues are shown in red, while positively charged residues are marked in blue. Protein domains I, II, III, and IV are labeled in yellow. The region of the *msp1* mutation (yellow square) is located in domain II. C and D show close-up views of the regions denoted by the yellow squares in panels A and B. The *msp1* mutation results in the loss of negative charge on the surface of the molecule. The bulky, negatively charged aspartic acid in the wild type protein is shown in red in panel C. This aspartic acid was replaced by glycine (a small, non-polar amino acid), shown in yellow in panel D.

To determine the mechanism by which the *msp1* mutation impairs the receptor function, we first examined structural models of mouse ERBB3 and the *msp1* mutant. Molecular modeling based on the solved three-dimensional structure of the human ERBB3 extracellular domain indicated that residue D313 is located in the CR1 of domain II. Introduction of the *msp1* missense mutation is not predicted to destabilize the structure or alter any pairwise interactions in the theoretical model (Figure 5A, B). However, the *msp1* substitution of glycine for aspartic acid at position 313 leads to a loss of negative charge on the molecule's surface (Figure 5C, D). The impact of this change on the local surface charge distribution, in a region distal to residues that interact with a ligand or are directly involved in receptor dimerization, is unclear.

**Figure 6:**
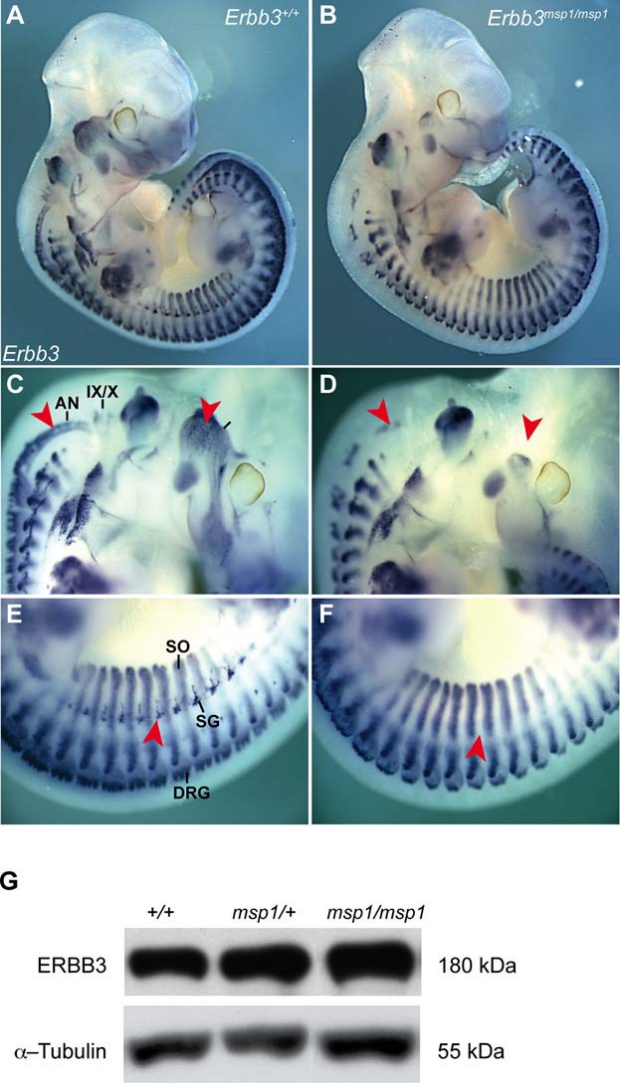
*Erbb3* mRNA and Protein Expression in *msp1* Mutants. Lateral view of *Erbb3* whole-mount *in situ* hybridization in control (A) and *Erbb3^msp1/msp1^* (B) embryos at E11.5. In control embryos, *Erbb3* transcript was detected in the cranial ganglia, cranial nerves (C), DRG, sympathetic ganglia (SG), and somites (SO) (E). The *Erbb3^msp1/msp1^* embryos showed absence of the *Erbb3* transcript in the trigeminal (V), superior (IX), and jugular (X) ganglia, accessory nerve (AN) and in the sympathetic chain (red arrowheads in D and F). *Erbb3* remained expressed in DRGs and somites in the *msp1* embryos. (G) Immunoblot analysis of the ERBB3 protein expression using the C-17 antibody specific for carboxy terminus of ERBB3 in *Erbb3^+/+^*, *Erbb3^msp1/+^* and *Erbb3^msp1/msp1^* whole-embryo lysates.

**Figure 7:**
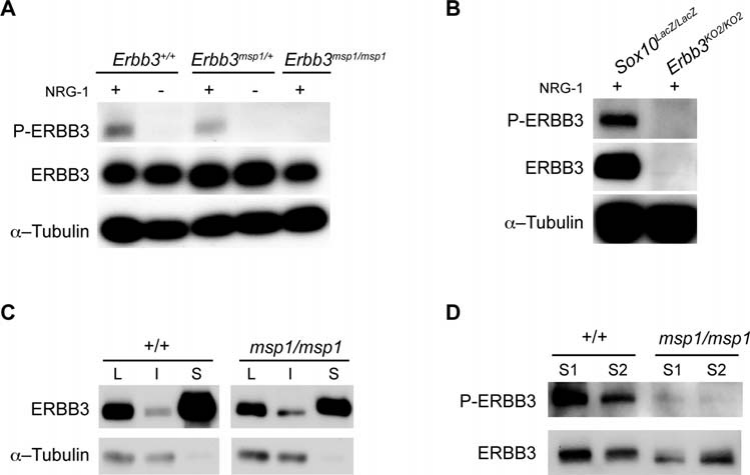
NRG1-β1-Induced Phosphorylation Is Impaired in ERBB3*^msp1/msp1^* Embryonic Cell Cultures. (A) Cells isolated from E11.5 embryos generated from *Erbb3^msp1^* heterozygous crosses were stimulated with or without NRG1-β1 (5 ng/ml) for 15 minutes, and cell extracts were collected. Equal amounts of proteins (20 ug) were separated on 8% SDS-PAGE gel, and membranes were blotted with the phospho-ERBB3, ERBB3 and α-Tubulin specific antibodies. NRG1-β1-induced phosphorylation of ERBB3 was detected in *Erbb3^+/+^* and *Erbb3^msp1/+^* cell cultures. In the absence of NRG1, no phosphorylation of ERBB3 was detected in these cells. *Erbb3^msp1/msp1^* cultures treated with NRG1-β1 revealed no band corresponding to the phosphorylated form of ERBB3. (B) Analysis of ERBB3 phosphorylation in *Sox10^LacZ/LacZ^* and *Erbb3^KO2/KO2^* embryonic cell cultures. In the presence of NRG1-β1, phosphorylation of ERBB3 was observed in *Sox10* mutant cultures, while no NRG1-β1-induced phosphorylation was detected in *Erbb3^KO2/KO2^* embryonic cells. (C) Cell surface biotinylation assay. ERBB3 was detected in total lysate (L), intracellular fraction (I) and surface fraction (S) in both wild-type and mutant embryonic cell cultures stimulated with NRG1-β1. No intracellular proteins were detected in the surface fraction of immunoprecipitates as indicated by anti-Tubulin immunoblotting. (D) Analysis of ERBB3 phosphorylation in avidin-precipitated fractions of embryonic cell cultures (1 mg of total lysates precipitated and loaded on the gel). Dramatically reduced ERBB3 phosphorylation was detected in the *msp1*cultures.

Since the results of structural modeling suggested no significant impact on receptor structure or function, we next evaluated the affect of the *msp1* mutation on *Erbb3* mRNA and/or protein expression. *Erbb3* mRNA expression was examined by *Erbb3* whole-mount *in situ* analysis (Figure 6 A–F). *In situ* hybridization revealed that *Erbb3* transcript was expressed in DRG and somites in *Erbb3^msp1/msp1^* mutants, but reduced in the cranial ganglia, cranial nerves, and sympathetic ganglia, indicating that the ENU mutation does not significantly alter *Erbb3* levels. Next, ERBB3 protein expression was evaluated by immunoblotting using an antibody that specifically recognizes the carboxy-terminus of the protein (C-17). A full-length ERBB3 protein (180 kDa) was observed in *Erbb3^msp1/msp1^* whole embryo lysates at E11.5 (Figure 6G). Lysates collected from *Erbb3^KO2/KO2^* embryos served as a negative control and showed no ERBB3 expression (Figure 7B). No significant differences in levels of protein expression were detected between control, *Erbb3^msp1/+^*, and *Erbb3^msp1/msp1^* lysates. All together, these results indicate that the mutation does not interfere with the *Erbb3* transcripts or protein translation.

### NRG1-β1-Induced Phosphorylation of *Erbb3* is Impaired in the *msp1* Embryos

To further examine the mechanism by which *Erbb3^msp1^* disrupts ERBB3 signaling, ligand-induced activation of ERBB3 was examined. Upon ligand binding, ERBB3 undergoes dimerization with a co-receptor (the most common being ERBB2), and this dimerization promotes phosphorylation of ERBB3 intracellular tyrosine residues. As phosphorylation of tyrosine residues is an indicator of the receptor's ability to become activated and is a requirement for the induction of downstream signaling, ERBB3 phosphorylation status was examined in *Erbb3^msp1/msp1^* embryonic cells. *Erbb3^msp1/+^* intercrosses were established, and embryos were collected at E11.5. Embryonic cells were isolated from whole E11.5 embryos, grown in culture for 24 hours and then treated with NRG1-β1 for 10 minutes. Upon NRG1-β1 stimulation, ERBB3 phosphorylation was detected in *Erbb3^+/+^* and *Erbb3^msp1/+^* cultures, while no ERBB3 phosphorylation was observed in *Erbb3^msp1/msp1^* cells (Figure 7A). This lack of ERBB3 tyrosine phosphorylation in *Erbb3^msp1/msp1^* cells could be due to altered ERBB3 function as a result of the *msp1* mutation, or could reflect the absence of cells that are able to respond to NRG1-β1 stimulus, such as the cranial and sympathetic ganglia. To test the latter hypothesis, we evaluated ERBB3 phosphorylation in *Sox10^LacZ^* homozygous mutants. *Sox10^LacZ^* homozygotes show reduced *Erbb3*-expressing cells in DRG, cranial and sympathetic ganglia, but *Erbb3* expression persists in somites (similar to *Erbb3* expression in somites and DRG in *Erbb3^msp1/msp1^* embryos, Figure 6B, D, and F) (Britsch et al., 2001). Analysis of ERBB3 tyrosine phosphorylation in *Sox10^LacZ^* homozygous cultures indicated that ERBB3 becomes phosphorylated in the presence of NRG1-β1 (Figure 7B), suggesting that the remaining *Erbb3*-expressing cells in *Sox10* mutants are sufficient to respond to NRG1-β1 stimuli. Therefore the absence of ERBB3 phosphorylation in the *Erbb3^msp1/msp1^* cultures is not due to a lack of NRG1-responsive cells, but rather to *msp1*-induced alteration of ERBB3 function.

To determine whether the *msp1* mutation prevents ERBB3 surface expression, we used a cell surface biotinylation assay on primary embryonic cell cultures. Embryonic cell cultures derived from several independent *Erbb3^msp1/+^* intercrosses were stimulated with NRG1-β1 and subsequently labeled with biotin. Wild-type and mutant cell lysates were immunoprecipitated with avidin-conjugated beads and further analyzed by Western blotting. When membranes were probed with the anti-ERBB3 antibody, the band corresponding to the ERBB3 protein was detected in the surface fraction of the *msp1* cultures (Figure 7C), indicating that ERBB3 is expressed on the cell membrane. Nonetheless, the band appeared slightly less intense when compared to wild-type. Analysis for tubulin in these samples validated the absence of intracellular protein biotinylation in the surface fraction (Figure 7C). Next, we evaluated the phosphorylation status of ERBB3 in the surface fraction using anti-phospho ERBB3 antibody. While ligand-induced phophorylation of ERBB3 was consistently detected in the avidin-precipitated fractions of wild-type cultures, only trace amounts of phosphorylation was observed in the *msp1* embryonic cells (Figure 7D). These results indicate that although the majority of ERBB3 is expressed on the cell surface, the mutant receptor is still insufficiently phosphorylated. Interestingly, when *Erbb3^msp1^* and *Erbb2* were transiently overexpressed in 293T cells, ERBB3 became phosphorylated in a ligand-dependent manner (Figure S2). Collectively, this suggests that *Erbb3^msp1^* is a hypomorphic allele and that forced overexpression of the mutant protein can overcome the severe reduction in ERBB3 phosphorylation that we observe *in vivo*.

## Discussion

### *Sox10* Expression Screen Identifies Loci Essential for NC Development

A whole-genome ENU mutagenesis screen was designed to identify mutations that alter patterning of *Sox10*-expressing NC derivatives. In the first 71 G_1_ lines analyzed, we mapped four heritable phenotypes that disrupt NC development. The frequency of phenotypes identified in our screen (6%) is consistent with previously published embryonic, phenotype-driven screens [26]–[30]. The variation in the frequency (3%–28%) reported in these screens is likely due in part to variations in the specificity of the phenotype being screened for as screens for gross morphology have produced a higher frequency of phenotypes than very focused, tissue specific screens.

One particular mutant line, *msp1*, identified in our screen, showed altered *Sox10^LacZ^* patterning only in the cranial and sympathetic ganglia. Expression analysis of *Sox10* and *Erbb3* demonstrated that DRG, enteric ganglia and melanocytes are not affected in the *msp1* mutants at E11.5, confirming that the ENU-induced mutation disrupts a specific subset of NC-derived cells. Through linkage analysis, sequencing, and complementation, we confirmed that the *msp1* phenotype is due to a point mutation in *Erbb3*, a member of the EGFR receptor tyrosine kinases family. The identification of the *Erbb3* allele in our *Sox10^LacZ^* expression screen indicates that we can successfully identify genes essential for *Sox10*-expressing cell lineages.

### *Erbb3^msp1^* Is a Novel Allele of *Erbb3*

Previously, two different null alleles of *Erbb3*, *Erbb3^KO1^* and *Erbb3^KO2^*, have been generated using gene-targeting technology [22]. *Erbb3^KO1^* mice produce truncated protein (∼120 kDa), while no protein is made from the *Erbb3^KO2^* allele. Animals homozygous for *Erbb3^KO1^* or *Erbb3^KO2^* allele are embryonic lethal due to cardiac malformation and show severe defects in development of the peripheral nervous system. In this study, we demonstrated that the *msp1* is a novel allele of *Erbb3*, *Erbb3^msp1^*, that carries a single amino acid substitution in the extracellular region of the protein. Unlike the null alleles, the full-length ERBB3 protein (∼180 kDa) is present in the *msp1* mutants. We showed that *msp1* failed to complement either of *Erbb3* null alleles, confirming that the *msp1* phenotype is indeed due to a molecular lesion in *Erbb3*. The onset of NC defect in *msp1* mutants is evident prior to E9.0, consistent with the data reported for *Erbb3* null mice. Furthermore, the *msp1* embryos die during gestation around E13.5 (Table S1), which is in accordance with the lethality data reported for *Erbb3^KO1^* and *Erbb3^KO2^* alleles [22]. While we could not detect any differences in phenotype between *msp1* and *Erbb3* null alleles in our assays, we cannot rule out the possibility that differences may occur in phenotypes not examined in this study. Nonetheless, our results indicate that the single amino acid change in *msp1* is sufficient to disrupt NC development and cause embryonic lethality without interfering with ERBB3 expression.

### How Does D313G Disrupt ERBB3 Function?

The finding that *Erbb3* mRNA and protein are present in *msp1* embryos, indicates that the ENU-induced mutation does not disrupt *Erbb3* transcription or translation. Comparative amino acid sequence analysis showed that the mutated amino acid (D313) is conserved among multiple species, suggesting that this residue could be critical for receptor activity. Indeed, biochemical analysis of receptor function revealed that ligand-induced phosphorylation of ERBB3 is disrupted in *Erbb3^msp1/msp1^* embryonic cell cultures (Figure 7A and D). Interestingly, we did observe ligand-dependent ERBB3 phosphorylation after transient co-expression of *Erbb3^msp1^* and *Erbb2* in 293T cells (Figure S2), indicating that the mutant ERBB3*^msp1^* is a severe hypomorphic allele that can become phosphorylated *in vitro*. As previous studies have shown, the difference in the phophorylation status could be attributed to differences in the efficiency of protein processing and post-translation modification in 293T cells compared to our embryonic cell cultures [31]–[33].

The loss of ligand-induced ERBB3 phosphorylation observed in the *msp1* embryos could be explained if the ERBB3 protein expressed in *msp1* embryos was not properly trafficked to the cell surface. However, our cell surface biotinylation assay showed that ERBB3 is expressed on the cell membrane in the *msp1* mutants. We consistently observed a small reduction in surface ERBB3 in the *msp1* embryonic cell cultures, implying that a small proportion of the mutant protein may not be properly folded and trafficked to the cell membrane. While additional studies are needed to conduct a more extensive analysis of the *msp1*-induced effect on ERBB3 stability and conformation, embryonic cell cultures are not the optimal system to address these questions due to the heterogeneous cell populations present in the wild-type and mutant cultures.

**Figure 8:**
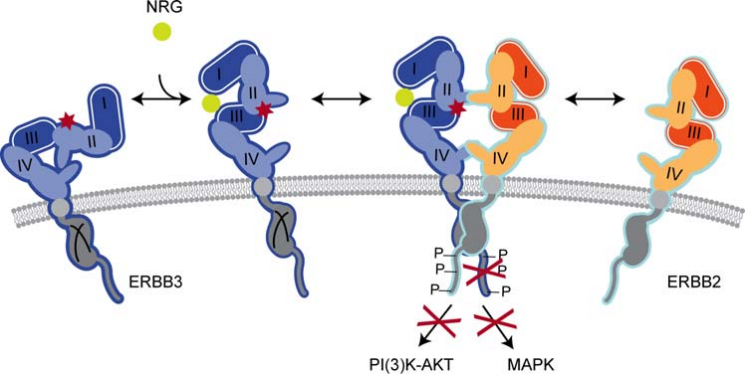
Proposed Mechanism by which the *msp1* Mutation Disrupts ERBB3 Function. In the absence of ligand (left), ERBB3 acquires a “tethered” conformation that renders the receptor inactive [36]. Upon ligand binding, the receptor undergoes a conformational change to allow dimerization with its binding partner, ERBB2 (right). Heterodimerization leads to autophosphorylation of ERBB2 and transphosphorylation of the kinase-dead ERBB3 receptor [34]. Phosphorylated tyrosine residues activate downstream signaling pathways, which elicit diverse cell responses. Ligand-induced phosphorylation of ERBB3 is impaired in the *msp1* (red Xs). The D313G mutation (red asterisk) may interfere with the receptors surface expression, ligand binding, and/or heterodimerization with ERBB2.

Although ERBB3 is expressed on the cell surface, it still remains insufficiently phosphorylated in the mutant embryos (Figure 7D). A second possibility that could contribute to impaired ERBB3 phosphorylation is that the D313G mutation might disrupt the ability of the ERBB3 protein to dimerize with its binding partner and/or bind to a specific ligand (Figure 8). ERBB3 lacks kinase activity due to evolutionary substitutions of critical residues within its catalytic domain [34], and thus can only signal within the context of a receptor heterodimer [35]. In the absence of NRG1, ERBB3 acquires a “tethered” conformation, which renders the receptor inactive. Previous structural studies proposed that, in this conformation, domains I and III are interlocked, thus preventing receptor dimer formation [36]. Furthermore, the dimerization arm, a ten-amino acid loop located in domain II (residues 242–259 of ERBB3), is bound to domain IV and, therefore, is unable to interact with another receptor. Upon ligand binding, the ERBB3 receptor undergoes a conformational change that allows for heterodimerization to occur. At the center of the dimer interface is a dimerization arm that reaches across and interacts with the dimerization arm of another receptor. The dimerization arm is conserved in all ERBB receptors, and deletions or mutations of these residues have been shown to completely abolish ligand-induced activation of EGFR [37],[38]. The *msp1* mutation is within domain II (CR1) of the extracellular region of the receptor. Since this domain is required for the receptor dimerization, one might predict that this mutation indirectly interferes with the ability of the receptor to dimerize with its dimerization partner. It is interesting to note that D313 is located in close proximity to E311 (D313 corresponds to E292 in the mature, processed protein) (Figure 5D). E311 of ERBB3 corresponds to EGFR residue E317 (E293 in the mature protein), and previous studies with EGFR have shown that upon ligand binding E317 forms a salt bridge with a residue in domain III, facilitating receptor dimerization [39]. Given the close proximity of D313 and E311 in ERBB3, it is possible that the D313G mutation interferes with salt bridge formation, thus destabilizing the interaction between domain II/III and preventing ligand-induced dimerization. However, we cannot exclude the possibility that the mutation may allosterically disrupt the ability of the receptor to bind to NRG1. While structural modeling provided no clear evidence of either effect, the inability of the ERBB3 receptor to bind to a ligand and/or to dimerize with its dimerization partner would be sufficient to disrupt ligand-induced phosphorylation and impair NRG1/ERBB3 signaling.

This study provides the first evidence of the importance of the aspartic acid residue at position 313 for ERBB3 receptor function. The identification of a novel *Erbb3* point mutation that alters NC development and leads to embryonic lethality demonstrates the feasibility of ENU-induced variations to help dissect the function of specific protein domains and provide information beyond what can be obtained from studying null alleles. Analysis of this and other mutant loci identified in our screen will shed additional functional insights into the molecular pathways governing NC development.

## Materials and Methods

### Mouse Husbandry

BALB/cJ and C57BL/6J inbred mouse strains were purchased from Jackson Laboratories (Bar Harbor, ME). Engineered mice with a LacZ cassette replacing the endogenous *Sox10* locus (*Sox10^LacZ/+^*; Britsch et al., 2001) were obtained on a mixed genetic background and maintained at NHGRI by crossing to C57BL/6J for several generations. All other mice described in the ENU screen were bred and housed in the NHGRI animal facility according to NIH guidelines. For genotyping, genomic DNA was prepared from tail biopsies or yolk sacs using a PUREGENE DNA purification kit (Gentra Systems, Inc. Minneapolis, MN) according to the manufacturers instructions. Noon on the day of vaginal plug observation was designated E0.5 for timed pregnancies. Reabsorbed embryos were counted as dead in analysis of lethality data.

### ENU Treatment and Mutagenesis Screen

ENU was prepared and injections were carried out as previously described [19].Briefly, ENU (Sigma; St. Louis, MO) was dissolved at 100 mg/ml in 95% ethanol and diluted to 5 mg/ml in a sterile phosphate/citrate buffer (0.1 M dibasic sodium phosphate, 0.05 M sodium citrate, pH 5.0). A spectrophotometer reading at a wavelength of 398 nm was used to confirm the ENU concentration, and BALB/cJ male mice were given weekly intraperitoneal injections at 0.1 mg per gram of body weight for three consecutive weeks. Mice were allowed to recover for eight weeks post-injection and loss of fertility was confirmed by mating to C57BL/6J females. Males that lost and subsequently regained fertility (G_0_) were bred to C57BL/6J females beginning at 12 weeks post-injection and the resulting first generation male progeny (G_1_) were crossed to *Sox10^LacZ/+^* females. In the next generation, female *Sox10^LacZ/+^* offspring (G_2_) were backcrossed to their G_1_ fathers, and embryos (G_3_) were harvested at E11.5 for β-galactosidase staining.

### Mapping

The number of samples and markers used in the initial genome scan for each pedigree is provided in Table 1. In each case DNA from affected G_3_ embryos and G_2_ obligate carriers was used for genotyping at simple sequence length polymorphism (SSLP) or single nucleotide polymorphism (SNP) markers spaced throughout the genome. The markers were polymorphic between the BALB/cJ and C57BL/6J parental strains used in this study and were genotyped by gel electrophoresis following PCR amplification (SSLPs) or using the Sentrix Universal Array Matrix platform (Illumina, San Diego, CA) (SNPs). Following the initial genome scan, additional markers from regions of significant linkage were used to refine the location of each *msp* locus and genotype additional G_2_ and G_3_ animals. Subsequently, markers flanking each locus (Table 1) were used to identify viable heterozygous carriers and maintain the line during six or more subsequent generations of outcrossing to C57BL/6J.

### DNA Sequencing

Using a candidate gene approach, *Erbb3* was chosen for sequencing in the *msp1* pedigree. Each exon and surrounding splice sites were amplified from genomic DNA for sequencing using standard techniques (Harvard Partners Healthcare Center for Genetics and Genomics, Harvard Medical School). The sequence of all *Erbb3* coding exons from an affected homozygote *msp1* mutant was compared to sequence from the parental inbred strains to identify a single nucleotide change on the ENU treated chromosome.

### TaqMan Assay for *msp1* Genotyping

Taqman MGB probes (Applied Biosystems, Foster City, CA) were designed across the site of the *msp1* mutation specific for both the wild-type allele (FAM-TGG-AAG-TAG-a-TAA-GAA-TG) and the ENU induced mutant allele (VIC-TGG-AAG-TAG-g-TAA-GAA-T). PCR reactions were carried out in 2× GenAmp PCR Master Mix, 10 µM each primer (primer 1: AGG-GCT-TGT-CCT-GCT-GAC-AA and primer 2: TGC-AAG-GCT-CAC-ACA-TCT-TGA), and 100 nM each allele-specific probe. Cycling conditions were 2 minutes at 50°C, 10 minutes at 95°C, followed by 40 cycles of 92°C for 15 seconds and 60°C for 1 minute. Relative quantitation of the two alleles was determined in an end-point assay for genotyping.

### β-Galactosidase Staining

Dissected embryos were fixed in 1× phosphate buffered saline (PBS), 1% formaldehyde, 0.2% glutaraldehyde, 0.02% NP-40 for 2 hours on ice, and X-gal staining was performed as previously described [40].

### Whole-Mount In Situ Hybridization

Whole-mount *in situ* hybridization was performed using digoxigenin-labeled antisense probes as previously described [41]. Reverse-transcribed digoxigenin-conjugated probes were made from linearized plasmids with polymerase binding site linkers (all reagents from Roche Diagnostic Incorporation, Indianapolis, IN). The following DNA sources were used for probe synthesis: 1.5-kb *Sox10* (nucleotides 1287–2787, dcgs10 *BssHII* T7 RNA polymerase), and *Erbb3* [42] (*EcoRI*, T7 RNA polymerase).

### Embryonic Cell Culture

Timed pregnancies between *Erbb3^msp1/+^* heterozygote mice were established and embryos were dissected out at E11.5. The yolk sac was stored for DNA isolation and used for genotyping of the *msp1* mutation. Dissected embryos were dissociated using a sterile razor blade, and digested in 0.05% Trypsin-EDTA for 30 minutes at 37°C. The trypsin digestion was inactivated with DMEM medium supplemented with 10% fetal bovine serum. To obtain single-cell suspension, digested embryos were pipeted up and down several times using a 2-ml pipet. The cell suspensions were transferred to a 15 ml conical tubes and spun down for 5 minutes at 1200×g. Supernatant was carefully removed, and cell pellet was resuspended in fresh DMEM medium supplemented with 10% fetal bovine serum, 10 nM Endothelin 3 (Sigma), 50 ng/ml Stem cell factor (R&D Systems, Minneapolis, MN) and 10 ng/ml Wnt-3a (R&D Systems) and cultured for 24 hours at 37°C and 5% CO_2_. The following day, cultures were stimulated with 5 ng/ml NRG1-β1 (R&D Systems) for 15 minutes and lysed in RIPA lysis buffer [1XTBS, 1% NP-40, 0.5% sodium deoxycholate, 0.1% SDS, 0.004% sodium azide] supplemented with 2 mM sodium orthovanadate, 4 mM phenylmethylsulfonyl fluoride, and protease inhibitor cocktail (Santa Cruz Biotechnology).

### Structural Modeling

Molecular modeling of the mouse ERBB3 extracellur region was performed using the MODELLER package [43] as implemented within Discovery Studio (Accelrys, San Diego, CA). Modeling was based on the solved structure of human ERBB3, as determined at a resolution of 2.6 Å by X-ray diffraction (pdb|1M6B) [36]. The mouse sequence was aligned with the human template sequence using Clustal W. The two sequences share 92% residue identity over the region modeled. To generate wild type and mutant structures, the MODELLER program was run in a fully-automated mode with a high optimization level in order to construct energy-minimized three-dimensional models of the aligned target sequences by satisfaction of spatial restraints extracted from the template PDB file.

### Immunoblot Analysis

Whole embryo lysates were prepared as follows. E11.5 embryos were dissected out and homogenized in RIPA lysis buffer supplemented with 1 mM Na_4_P_2_O_7_, 2 mM PMSF and protease inhibitor cocktail (Santa Cruz Biotechnology, Santa Cruz, CA) followed by a brief sonication. The lysates were cleared by centrifuged at 14,000×g for 10 minutes at 4°C, and protein concentration of the supernatant was determined using a BCA protein assay kit (Pierce, Rockford, IL). Cell lysates containing equal amounts of protein were separated on 8% Tris-Glycine SDS gel (Invitrogen, Carlsbad, CA), transferred to polyvinylidene fluoride membrane (Invitrogen) and blocked with 5% milk in TBST [20 mM Tris-HCl (pH 7.5), 150 mM NaCl, 0.05% Tween 20] for 1 hour at room temperature. The membranes were then incubated with primary antibody against ERBB3 (1∶1000 diluted in 5% milk in TBST) (Santa Cruz Biotechnology; C-17), phospho-ERBB3 (1∶500 diluted in 5% bovine serum albumin (BSA) in TBST) (Cell Signaling, Santa Cruz, CA; 21D3), α-Tubulin (1∶1000 diluted in 5% milk in TBST) (Sigma) at 4°C over night. After washing in TBST, the membranes were incubated with horseradish peroxidase-conjugated secondary antibodies (1∶10,000) (Amersham Biosciences, Little Chalfont, UK) for 1 hour at room temperature. The protein bands were detected using an enhanced chemiluminescence kit (Millipore, Billerica, MA).

### Cell Surface Biotinylation Assay

Embryonic cell cultures were prepared as described above. On day two, cultures were stimulated with NRG1-1β for 10 minutes, washed twice in 1× PBS and chilled on ice for 5–10 minutes. Cell surface proteins were labeled with 1 mg/ml EZ-Link Sulfo-NHS-SS-Biotin (Pierce, Rockford, IL) in PBS for 30 minutes at 4°C with gentle shaking. Biotin was aspirated and cultures were washed twice for 3–5 minutes with 100 mM Tris, pH 8.0 in PBS followed by one wash in 1× PBS. Cells were solubilized in mild-lysis buffer (20 mM Tris, pH 7.4, 150 mM NaCl, 1 mM MgCl_2_, 1% NP-40, 10% glycerol) supplemented with 1 mM Na_4_P_2_O_7_, 2 mM PMSF and protease inhibitor cocktail (Santa Cruz Biotechnology, Santa Cruz, CA) for 30 minutes at 4°C on a shaker, and centrifuged for 15 minutes at 10,000×g. Supernatants were removed and the protein concentration determined by a BCA protein assay kit (Pierce, Rockford, IL). An equal amount of protein (1 mg) of each genotype was precipitated with 100 ul NeutrAvidin agarose beads (Pierce, Rockford, IL) overnight at 4°C with rotation. Beads were washed three times in mild-lysis buffer, and surface proteins were eluted in 50 ul 2× SDS loading buffer supplemented with 50 mM DTT (Invitrogen, Carlsbad, CA). Equal volumes (40 ul) of each genotype were separated on 8% Tris-Glycine SDS gel, and membranes were blotted with anti-ERBB3 and/or phospho-ERBB3 antibody.

### Site-Directed Mutagenesis

The *msp1* mutation was introduced in the human *Erbb3* cDNA (Clone ID #6147464, Open Biosystems, Huntsville, AL) via QuickChange II Site-Directed Mutagenesis Kit according to manufacturer's instructions (Stratagene, La Jolla, CA). The following PCR primers were used to introduce the mutation: Fwd-CCTGACAAGATGGAAgTAGGTAAAAATGGGCTCAAGATG- and Rev-CATCTTGAGCCCATTTTTACCTACTTCCATCTTGTCAGG-. The presence of the *msp1* mutation was confirmed by sequencing.

### Cell Culture and Transfection

293T cells were cultured in DMEM medium with 10% fetal bovine serum, 100 units/ml penicillin and 100 ug/ml streptomycin at 37°C in a humidified 5% CO_2_. *Erbb3^msp1^* and *Erbb2* (Clone ID #6178526, Open Biosystems) or wild-type *Erbb3* and *Erbb2* clones were co-expressed in 293T cells using FuGENE 6 (Roche Applied Science) transfection reagent. 48 hours post-transfection, cells were starved overnight in serum-free DMEM. The following day, cells were stimulated with or without 5 ng/ml NRG1- β1 (R&D Systems) for 15 minutes. Cell lysates were collected in RIPA lysis buffer and analyzed by Western blotting.

## Supporting Information

Figure S1 Schematic representation of the embryonic ENU screen for the recessive mutations that alter *Sox10^LacZ^* expression. ENU treated BALB/C males are mated with C57BL/6J female to generate G_1_ offspring. G1 males are further crossed with *Sox10^LacZ/+^* females to obtain G_2_ progeny. G2 *Sox10^LacZ/+^* females are backcrossed to G_1_ mutagenized males. The resulting G_3_ embryos are collected at E11.5, stained for β-galactosides activity, and analyzed for altered *Sox10^LacZ^* expression.
(3.47 MB TIF)

Figure S2 Ligand-induced Phosphorylation of *Erbb3^msp1^* in 293T Cells. 293T cells were cotransfected with *Erbb2* and *Erbb3* or with *Erbb2* and *Erbb3^msp1^* cDNA. Subsequently, cells were stimulated with NRG1-β1 and harvested for immunoblot analysis. NRG1-β1-induced phosphorylation of ERBB3 was detected and compared between cells transfected with wild-type ERBB3 and cells transfected with the *msp1* mutant ERBB3.
(0.1 MB TIF)

Table S1 Embryonic and postnatal viability of the *msp1* mutants.
(0.03 MB DOC)
