# Supplementary figures and images for: A Sox10 Expression Screen Identifies an Amino Acid Essential for Erbb3 Function
Source: PLoS Genet. 2008 Sep 5;4(9):e1000177. doi: 10.1371/journal.pgen.1000177 (PMC2518866; doi:10.1371/journal.pgen.1000177)

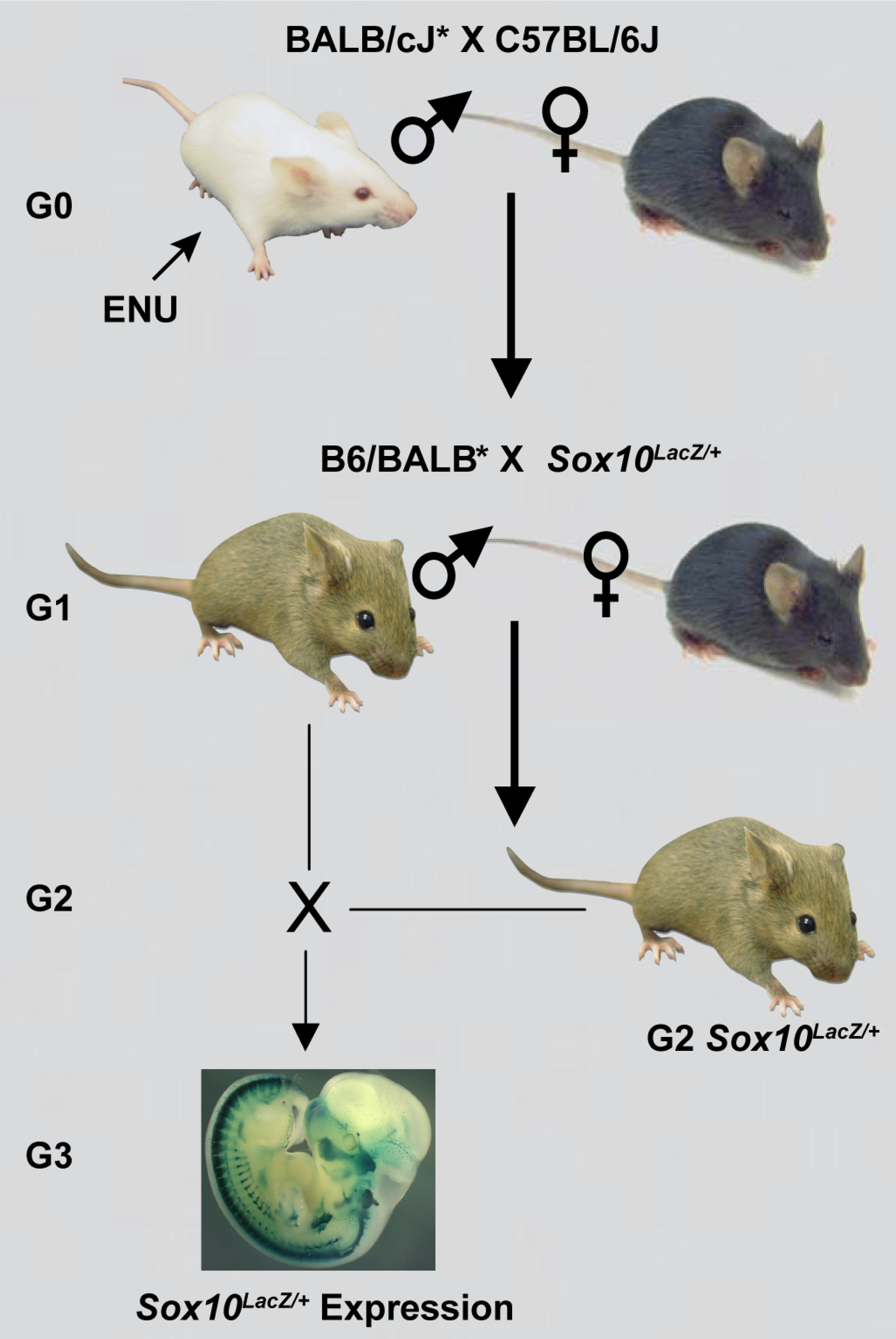

Supplement: Figure S1 — Schematic representation of the embryonic ENU screen for the recessive mutations that alter Sox10LacZ expression. ENU treated BALB/C males are mated with C57BL/6J female to generate G1 offspring. G1 males are further crossed with Sox10LacZ/+ females to obtain G2 progeny. G2 Sox10LacZ/+ females are backcrossed to G1 mutagenized males. The resulting G3 embryos are collected at E11.5, stained for β-galactosides activity, and analyzed for altered Sox10LacZ expression. (3.47 MB TIF) [file pgen.1000177.s001.tif]

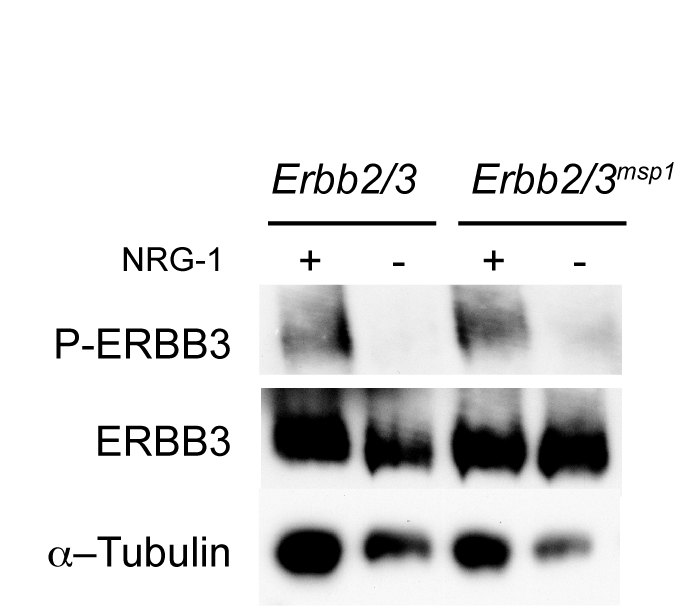

Supplement: Figure S2 — Ligand-induced Phosphorylation of Erbb3msp1 in 293T Cells. 293T cells were cotransfected with Erbb2 and Erbb3 or with Erbb2 and Erbb3msp1 cDNA. Subsequently, cells were stimulated with NRG1-β1 and harvested for immunoblot analysis. NRG1-β1-induced phosphorylation of ERBB3 was detected and compared between cells transfected with wild-type ERBB3 and cells transfected with the msp1 mutant ERBB3. (0.1 MB TIF) [file pgen.1000177.s002.tif]
