# Supplementary material for: A Sox10 Expression Screen Identifies an Amino Acid Essential for Erbb3 Function
Source: PLoS Genet. 2008 Sep 5;4(9):e1000177. doi: 10.1371/journal.pgen.1000177 (PMC2518866; doi:10.1371/journal.pgen.1000177)
Supplement: Table S1 — Embryonic and postnatal viability of the msp1 mutants. (0.03 MB DOC) [file pgen.1000177.s003.doc]

| **Supplemental Table 1: Embryonic and postnatal viability of the *msp1* mutants** | | | |
| --- | --- | --- | --- |
|  | Number of viable embryos | | |
| Age | *ErbB3^+/+^* | *ErbB3^msp1/+^* | *ErbB3^msp1/msp1^* |
| E11.5 | 28 | 47 | 25 (25%) |
| E12.5 | 33 | 40 | 15 (17%) |
| E13.5 | 25 | 42 | 6 (8%) |
| E16.5 | 12 | 21 | 1 (3%) |
| At weaning | 27 (35%) | 51 (65%) | 0 |
